# Reliability of DIERS pedogait system for evaluating spatiotemporal gait parameters in knee osteoarthritis and its association with Achilles tendon stiffness asymmetry

**DOI:** 10.3389/fphys.2026.1780014

**Published:** 2026-02-11

**Authors:** Xingxing Shen, Jiahao Chen, Jiahao Zhang, Jiaqing Tian, Sirun Cheng, Jichuan Cao, Congcong Li, Xuemeng Xu

**Affiliations:** 1 The Fifth Clinical School of Medicine, Guangzhou University of Chinese Medicine, Guangzhou, Guangdong, China; 2 The First Affiliated Hospital of Zhejiang Chinese Medical University, Hangzhou, Zhejiang, China; 3 Guangdong Second Traditional Chinese Medicine Hospital, Guangzhou, Guangdong, China; 4 Guangdong Provincial Key Laboratory of Research and Development in Traditional Chinese Medicine, Guangzhou, Guangdong, China

**Keywords:** Achilles tendon biomechanics, asymmetry, clinical assessment, DIERS pedogait system, gait analysis, knee osteoarthritis, reliability

## Abstract

**Objective:**

To assess the inter-rater and test-retest reliability of DIERS pedogait system for measuring gait parameters in patients with knee osteoarthritis (KOA), and to explore inter-limb differences in Achilles tendon (AT) properties, as well as the associations between the AT stiffness asymmetry index (Asy_Stiffness_ (AT)) and gait abnormalities, visual analog scale (VAS) scores, and Kellgren-Lawrence (K/L) grades.

**Methods:**

A total of 36 patients with KOA (19 unilateral, 17 bilateral) were enrolled. Two independent assessors used DIERS pedogait system to measure gait parameters, with retesting by the first assessor 1 week later. Inter-rater and test-retest reliability were quantified using intraclass correlation coefficients (ICC), while absolute reliability was assessed using standard error of measurement (SEM), minimum detectable change (MDC), and Bland-Altman analysis. Bilateral AT muscle tone and stiffness were evaluated using the MyotonPRO. Spearman correlation and multiple linear regression analyses were performed to explore the associations of AsyStiffness (AT) with both clinical variables and gait parameters.

**Result:**

DIERS pedogait system exhibited excellent inter-rater (ICC: 0.900–0.987) and test-retest reliability (ICC: 0.927–0.988). Inter-rater SEM and MDC ranged from 0.07 to 44.26 and 0.20 to 122.68, respectively, while test-retest SEM and MDC ranged from 0.05 to 39.59 and 0.13 to 109.74. Bland-Altman analysis revealed no significant systematic bias. In addition, AT muscle tone and stiffness were significantly higher in the relatively severe leg (RSL) compared with the moderate leg (RML) (*P* < 0.05). Asy_Stiffness_ (AT) was positively correlated with stance phase symmetry index (*ρ* = 0.514, *P* = 0.001), stride time (*ρ* = 0.381, *P* = 0.022), VAS score (*ρ* = 0.373, *P* = 0.025), and K/L grade (*ρ* = 0.542, *P* = 0.001), and negatively correlated with gait speed (*ρ* = −0.374, *P* = 0.025). Multiple linear regression identified stance phase symmetry index (*β′* = 0.298, *P* = 0.043), K/L grade 2 (*β′* = 0.533, *P* = 0.017) and K/L grade 3 (*β′* = 0.778, *P* = 0.002) as independent factors associated with Asy_Stiffness_ (AT).

**Conclusion:**

DIERS pedogait system is a reliable and objective tool for assessing gait in KOA patients. AT stiffness asymmetry is associated with gait abnormalities, pain, and KOA severity. These findings suggest that early-mid interventions targeting gait abnormalities and mitigating Achilles tendon stiffness asymmetry may provide novel prophylactic and therapeutic strategies for KOA.

## Introduction

Knee osteoarthritis (KOA) is a prevalent chronic degenerative joint disease, primarily characterized by pain, swelling, joint deformities, and functional impairment (Katz et al., 2021; Arden et al., 2021). KOA is a leading cause of lower limb disability among the elderly, with prevalence estimates reaching up to 3.8% of the population, and a higher incidence observed in women (Avendaño-Coy et al., 2020). With population aging, the incidence of KOA continues to rise, placing a substantial burden on public health systems and social economy (Cui et al., 2020). The etiology of KOA is multifactorial, with contributing factors including obesity, aging, gender, knee joint trauma, and genetic predisposition (Briggs-Price et al., 2022; Blagojevic et al., 2010). Addressing this pressing health issue requires a comprehensive understanding of its underlying pathogenesis, to inform both preventive strategies and therapeutic interventions. Recent studies have increasingly identified that the progression of KOA is closely related to the biomechanical changes in the lower extremities (Shen et al., 2025; Chen et al., 2021).

Abnormal gait is a prevalent manifestation of lower limb dysfunction in KOA patients. To mitigate knee joint pain, KOA patients often adopt compensatory gait patterns that reduce the load on the affected joint (Fukaya et al., 2019). Common adjustments include a reduction in walking speed, shortened stride length, increased cadence, and prolonged support and double-support phases (Messier et al., 2016; Mills et al., 2013). Although these compensatory changes may temporarily alleviate pain, existing research has confirmed that long-term gait asymmetry is associated with alterations in knee joint load distribution and accelerated structural degeneration (Creaby et al., 2012). Previous studies have demonstrated that asymmetric gait is highly prevalent in both healthy individuals and KOA patients (Lathrop-Lambach et al., 2014; Messier et al., 2016). Therefore, accurate and reliable assessment of gait characteristics is critical for understanding the biomechanical mechanisms of KOA, monitoring disease progression, and evaluating the efficacy of treatments.

Various clinical assessments, such as the Timed Up and Go (Iijima et al., 2018) and the Stair Climb Test (Holm et al., 2022), are widely used to evaluate gait abnormalities in KOA patients. These measures are simple to administer, inexpensive, and well suited for rapid screening. However, their main limitation is that they do not directly quantify gait asymmetry or detailed spatiotemporal gait parameters. Three-dimensional (3D) gait analysis is regarded as the gold standard in gait assessment (D'Souza et al., 2024; Scataglini et al., 2024), which offers precise measurements of biomechanical features such as joint angles, step length, step speed, and joint moments. Despite its accuracy, this technique is hindered by the high cost of equipment, operational complexity, and the need for specialized personnel and facilities, thus limiting its broader clinical application. In contrast, DIERS pedogait system is non-invasive, user-friendly, and time-efficient, automatically capturing spatiotemporal gait parameters via a treadmill. Previous studies have established the reliability of this system for assessing spinal morphology and cervical motion in healthy individuals (Degenhardt et al., 2020; Mangone et al., 2018). However, it has not yet been widely adopted for assessing abnormal gait in KOA patients. It is of paramount significance to establish and verify the reliability of DIERS pedogait system for evaluating gait abnormalities in KOA patients.

An expanding body of evidence indicates that the progression of KOA is not solely attributed to the knee joint itself, but is also closely linked to functional alterations in adjacent joints and muscles (Chen et al., 2021). The Achilles tendon is one of the largest and most important tendons in the human body and a key connection between the calf muscles and the calcaneus (Meredith et al., 2025; Nazir and Ansari, 2024). Its influence on lower limb biomechanics extends beyond the ankle joint, impacting the function and alignment of neighboring structures, including the knee (Elbaz et al., 2017). Studies have revealed a significant correlation between Achilles tendon thickness and the severity of KOA (Elbaz et al., 2017). Furthermore, researchers have shown that decreased plantarflexor strength and abnormal gait patterns (such as reduced gait speed) are recognized risk factors for Achilles tendinopathy (Liang et al., 2024). These changes may alter the muscle properties of the Achilles tendon, such as muscle tone and stiffness. These alterations in muscle properties can be assessed using a non-invasive digital palpation device called MyotonPRO, which facilitates rapid and precise assessment of superficial muscle properties (Aird et al., 2012).

It is reported that KOA patients commonly exhibit unilateral or bilateral asymmetry in the properties of the Achilles tendon (Chen et al., 2021). However, the factors contributing to this asymmetry remain poorly understood. Given that KOA patients often exhibit concurrent gait abnormalities and alterations in Achilles tendon properties, we hypothesize a potential correlation between these two variables. Accordingly, our study aims to establish the reliability of DIERS pedogait system for assessing gait abnormalities in patients with KOA. Additionally, it also seeks to explore the differences in the properties of the Achilles tendon between different limbs in KOA patients, and examine the relationship between this asymmetry (including muscle tone and stiffness) and gait parameters, visual analog scale (VAS) score, and Kellgren-Lawrence (K/L) grade. These findings will enable precise assessment of symptom severity in KOA patients, thereby establishing a new theoretical basis for the formulation of prevention and treatment strategies for KOA.

## Methods

### Study design

The study was conducted at the Orthopedic Clinic of Guangdong Second Hospital of Traditional Chinese Medicine from September 2022 to June 2024. The protocol was approved by the Ethics Committee of the Second Hospital of Traditional Chinese Medicine of Guangdong Province (No. 2021(K58)) and registered with the Chinese Clinical Trial Registry (ChiCTR2100050269; registration date: 25 August 2021). Written informed consent was obtained from all participants, and all methods were carried out in accordance with relevant guidelines and regulations.

### Participants

The sample size was calculated using the PASS 15.0.5 software. It was derived through an estimation method from a previous reliability study (Zou, 2012). This approach aimed for an 80% probability that the lower bound of the 95% confidence interval of the intraclass correlation coefficient (ICC) would not be less than 0.7, assuming an expected ICC of 0.8. The calculation indicated that at least 25 participants were required. Inclusion criteria: (1) Patients with unilateral or bilateral knee osteoarthritis diagnosed according to the American College of Rheumatology clinical criteria, defined by knee pain plus at least three of the following six features: age >50 years, morning stiffness <30 min, crepitus, bony tenderness, bony enlargement, and no palpable warmth (Altman et al., 1986); (2) Age between 50 and 75 years; (3) Kellgren-Lawrence grade ≥1 in at least one knee (Kellgren and Lawrence, 1957); (4) Body mass index (BMI) between 18 and 30 kg/m^2^; (5) Ability to complete the gait test without assistive devices or external assistance. Exclusion criteria: (1) Presence of other inflammatory arthritis, such as rheumatoid arthritis; (2) Presence of neurological disorders, such as stroke, spinal-related diseases, Parkinson’s disease, severe cardiovascular or respiratory diseases, or other musculoskeletal diseases; (3) had taken analgesic drugs in the past month; (4) Congenital or traumatic lower-limb deformity; (5) Inability to return for repeat testing; (6) had engaged in vigorous exercise within 48 h before the study.

For the included KOA patients, legs with higher K/L grades were classified as relatively severe legs (RSL), while those with lower K/L grades were categorized as relatively moderate legs (RML). In cases where both legs exhibited the same K/L grade, the leg with the higher pain score on the visual analogue scale (VAS) was designated as the RSL, and the leg with the lower score was assigned to the RML category. In instances where bilateral KOA patients had identical K/L grades and VAS scores, the classification of RSL and RML was determined randomly (e.g., coin tossing).

### Evaluation steps of gait

The gait of the participants was measured using a DIERS pedogait analyzer (DIERS International GmbH, Schlangenbad, Germany, Model: formetric) (Supplementary Figure S1). Assessments were performed by two independent raters (XX-S and CC-L). Both raters participated in DIERS system operation training course and underwent standardized calibration to familiarize themselves with each item in the assessment tool, aiming to minimize inter-rater differences. During the assessment period, participants were required to walk barefoot and in a natural, relaxed state at a constant speed on the treadmill of DIERS Gait Analysis System for approximately 10 min, to allow them to gradually adapt to the speed of the treadmill. After the participants gradually attained a steady walking state, they were instructed to maintain the target speed for a continuous 5-min interval; during the middle 30 s of this period, testers collected gait data. Participants were required to look straight ahead, not to touch the handrail, and do not take large steps during the assessment (Wang et al., 2024). After the first assessor (XX-S) completed all measurements, participants were required to rest for 10 min before the second assessor (CC-L) repeated the gait assessment using the same protocol. Inter-rater reliability was calculated from the two assessors’ measurements. One week later, the first assessor (XX-S) re-evaluated all participants using the same procedure to assess test–retest reliability. Throughout the entire assessment procedure, the two assessors remained blinded to evaluations conducted by one another, and neither assessor had access to the demographic and clinical characteristics of the participants or their specific pain sites. Each participant performed three measurements, and the mean value was taken as the final result. A gait cycle was defined as the process from the touch of the right heel to the second touch of the right heel. After the measurement was completed, we collected the following test indicators: (1) Temporal parameters: single support phase (%), double support phase (%), stance phase (%), swing phase (%), stride time (ms), and step time (ms); (2) Spatial parameters: gait speed (km/h), step length (cm), stride length (cm), and step width (cm).

Furthermore, building on the results of previous analogous studies, gait data derived from the left and right feet were pooled for reliability and correlation analyses (Evans et al., 2003).

### Evaluation of gait symmetry index

Employing the symmetric index (SI) as defined in prior work, we performed a comparative analysis of gait variables across both lower limbs of the participants. This equation determines the asymmetry between limbs, unaffected by the side (Herzog et al., 1989).

$$SI=\frac{\left|V_{L}-V_{R}\right|}{\frac{1}{2}\left(V_{L}+V_{R}\right)}\times 100\%$$

$V_{L}$ denotes gait variables for the left lower limb, and $V_{R}$ represents those for the right lower limb. An SI value of 0 indicates perfect bilateral symmetry, whereas values of SI ≤10% signify clinically acceptable asymmetry (Messier et al., 2016). The absolute value function quantifies the magnitude of asymmetry, irrespective of the association between RSL and RML.

### Evaluation of properties of achilles tendon

One team member (JH-C) was randomly assigned to quantify the properties of the Achilles tendon (AT) using a portable MyotonPRO device (MyotonPRO, Estonia, serial number: 000,297, product manufacturer code: 1308600502). The team member underwent standardized training on device operation and attained proficiency in MyotonPRO usage. Based on prior studies (Huang et al., 2018; Chen et al., 2021), AT was measured at 6 cm from bone insertion (Chen et al., 2021) (Supplementary Figure S2). During testing, participants were required to lie on the treatment bed with their lower legs relaxed. One researcher (XX-S) kept the participant’s knee fully extended and positioned the ankle at approximately 45° of plantar flexion, while another researcher (JH-C) placed the MyotonPRO probe perpendicular to the skin over the marked AT site. When adequate probe pressure was achieved (signaled by the device’s light turning from red to green), the device delivered five rapid mechanical impulses to the tendon, completing one measurement cycle. During the measurement, the instrument first applied a preload of 0.18N to the soft tissue, then generated an impulse of 0.58N over 15 ms to induce a damped oscillation in the muscle tissue. According to the oscillation signal, the device calculated the oscillation frequency (F; Hz) and stiffness (S; N/m). Following completion of measurements, the instrument automatically computes and displays the coefficient of variation (CV) for each parameter, in accordance with the standard statistical formula below:

$$CV\;\left(\%\right)=\frac{Standard\;Deviation\;\left(SD\right)}{Mean\;Value\;\left(\mu \right)}\times 100\%$$

If CV exceeds 3%, retesting is required (Núñez et al., 2023). Muscle tone and stiffness values were averaged across three measurement cycles.

### Calculation of achilles tendon properties asymmetry indexes

According to previous research methods (SupplementaryMethod 1), we calculated asymmetry indexes for Achilles tendon muscle tone and stiffness. Larger index values correspond to greater interlimb imbalances in muscle function (Chen et al., 2021). The Achilles tendon muscle tone asymmetry indexes and stiffness asymmetry indexes were denoted as Asy_Tone_ (AT) and Asy_Stiffness_ (AT).

### Statistical analysis

Statistical analyses were performed using SPSS Version 26.0 (IBM, Corp., NY, United States). Categorical variables were summarized as counts and percentages. Continuous data were tested for normality using the Shapiro-Wilk test. Normally distributed measurements were expressed as mean ± standard deviation (SD), whereas non-normally distributed data were expressed as median and interquartile range (IQR). Levene’s test was used to verify the homogeneity of variances. Paired sample t-test was applied to compare RSL versus RML measurements within the same patients when data were normally distributed, whereas a nonparametric Wilcoxon signed-rank test was used for paired comparisons of non-normally distributed data. Inter-rater reliability was assessed with a two-way random model (average measures), and test-retest reliability was assessed with a two-way mixed model (single measures). For each outcome measure derived from DIERS pedogait, the ICC was calculated to evaluate relative reliability. ICC values range from 0 to 1, with values <0.40 indicating poor reliability, 0.40–0.59 indicating moderate reliability, 0.60–0.74 good reliability, and ≥0.75 excellent reliability (van Geel et al., 2020). Absolute reliability metrics were also cocalculated, including the standard error of measurement (SEM), minimal detectable change (MDC), and 95% limits of agreement (LOA) (Wang et al., 2023). These indicators were derived using the formulas: SEM = SD × √(1 – ICC); MDC = 1.96 × SEM × √2 (Weir, 2005); and 95% LOA = mean difference ±1.96 × SD. A lower SEM corresponds to higher measurement precision, and the MDC represents the smallest change in a value that exceeds the expected measurement error. Bland–Altman plots (MedCalc version 22.0, Ostend, Belgium) were used to assess systematic bias between measurements for both inter-rater and test-retest comparisons (Bland and Altman, 2007). Spearman’s correlation coefficient was used to evaluate the associations between the asymmetry index of the AT muscle properties (muscle tone and stiffness) and the parameters of DIERS treadmill, VAS, and K/L grade. Correlation strength was defined as >0.70 indicating a “strong” correlation, 0.50–0.70 “good,” 0.30–0.50 “moderate,” and <0.30 “poor” (Hazra and Gogtay, 2016). A correlation coefficient greater than 0.30 was deemed to indicate acceptable consistency between measures (Koo and Li, 2016). Independent factors influencing the properties of the Achilles tendon were identified using multiple linear regression analysis. Variance inflation factor (VIF) values quantify the extent of multicollinearity among independent variables, with the following general thresholds: VIF <5 indicating no significant multicollinearity, 5 ≤ VIF <10 indicating moderate multicollinearity, and VIF ≥10 indicating severe multicollinearity. Statistical significance was defined as *p* < 0.05.

## Result

### Participants characteristics

**FIGURE 1:**
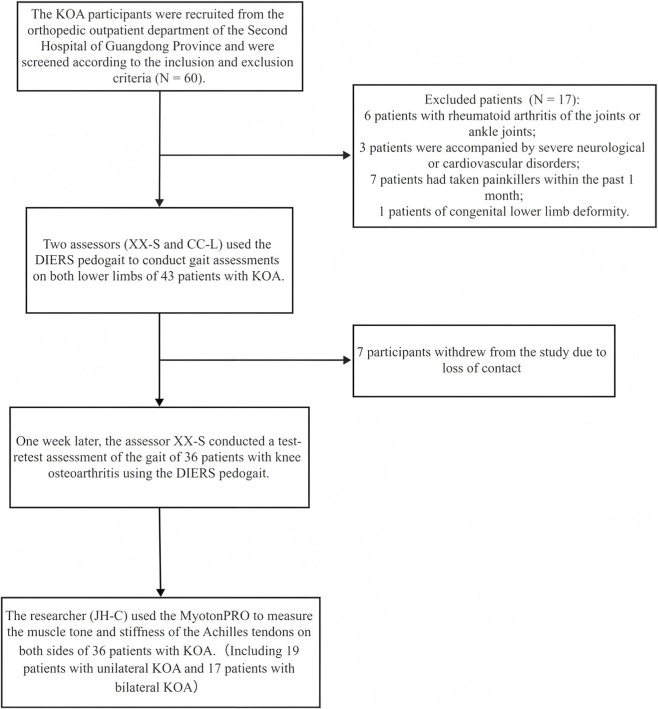
The screening and testing process for KOA participants.

Following the inclusion and exclusion criteria, a total of 36 patients with unilateral or bilateral KOA were enrolled, including 19 patients with unilateral pain and 17 patients with bilateral pain. Participant demographics and foot morphological parameters were summarized in Table 1, and the screening and testing process for KOA participants was shown in Figure 1.

**TABLE 1:** Participant characteristics (n = 36).

Variable	Mean ± SD or n (%)
Age (years)	62.97 ± 10.08
Gender (female/Male)	29/7
Height (cm)	162.19 ± 7.22
Weight (kg)	59.46 ± 8.50
BMI (kg/m^2^)	22.60 ± 2.86
Kellgren-lawrence classification	
Grade 1	3 (8.3%)
Grade 2	12 (33.3%)
Grade 3	20 (55.6%)
Grade 4	1 (2.8%)
VAS (RSL)	5.00 (4.25, 7.00)
Limb involvement	
Unilateral	19 (52.8%)
Bilateral	17 (47.2%)
RSL (left/right)	14/22
RML (left/right)	22/14

SD, standard deviation; BMI, body mass index; VAS, visual analogue scale; RSL, relatively severe leg; RML, relatively moderate leg.

### Inter-rater reliability

The gait parameters collected by the raters are presented in the Supplementary Material (Supplementary Table S1). Table 2 summarized the inter-rater consistency of 72 limb gait parameters measured in 36 patients. The ICC values ranged from 0.900 to 0.987, with corresponding 95% confidence intervals spanning 0.803 to 0.993. The SEM across gait parameters ranged from 0.07 to 44.26, while the MDC values ranged from 0.20 to 122.68. These findings indicated excellent inter-rater reliability for all gait parameters, as shown in Table 2.

**TABLE 2:** Inter-rater reliability of gait parameters.

Variable	SD	ICC (95% CI)	SEM	MDC	P Value
Temporal parameters					
Single support phase (%)	2.86	0.952 (0.924, 0.970)	0.63	1.74	<0.01
Double support phase (%)	2.82	0.942 (0.907, 0.964)	0.68	1.88	<0.01
Stance phase (%)	2.19	0.946 (0.914, 0.966)	0.51	1.41	<0.01
Swing phase (%)	2.01	0.956 (0.913, 0.977)	0.42	1.17	<0.01
Stride time (ms)	81.09	0.965 (0.945, 0.978)	15.17	42.05	<0.01
Step time (ms)	139.96	0.900 (0.803, 0.949)	44.26	122.68	<0.01
Spatial parameters					
Step length (cm)	6.45	0.987 (0.979, 0.992)	0.74	2.04	<0.01
Stride length (cm)	13.23	0.943 (0.888, 0.971)	3.16	8.76	<0.01
Step width (cm)	3.47	0.986 (0.972, 0.993)	0.41	1.14	<0.01
Gait speed (km/h)	0.25	0.920 (0.843, 0.959)	0.07	0.20	<0.01

SD, standard deviation; ICC, intraclass correlation coefficient; 95% CI, 95% confidence intervals; SEM, standard error of measurement; MDC, the minimal detectable change at a 95% confidence level.

### Test–retest reliability

Table 3 summarized the test–retest consistency of 72 limb gait parameters measured in 36 patients. The ICC values ranged from 0.927 to 0.988, with corresponding 95% confidence intervals spanning 0.862 to 0.994. The SEM across gait parameters ranged from 0.05 to 39.59, while the MDC values ranged from 0.13 to 109.74. These findings indicated excellent test–retest reliability for all gait parameters, as shown in Table 3.

**TABLE 3:** Test–retest reliability of gait parameters.

Variable	SD	ICC (95% CI)	SEM	MDC	P Value
Temporal parameters					
Single support phase (%)	2.87	0.969 (0.950, 0.980)	0.51	1.40	<0.01
Double support phase (%)	2.75	0.979 (0.966, 0.987)	0.40	1.10	<0.01
Stance phase (%)	2.18	0.960 (0.938, 0.975)	0.44	1.21	<0.01
Swing phase (%)	2.05	0.978 (0.958, 0.989)	0.30	0.84	<0.01
Stride time (ms)	80.38	0.972 (0.956, 0.982)	13.45	37.28	<0.01
Step time (ms)	146.54	0.927 (0.862, 0.962)	39.59	109.74	<0.01
Spatial parameters					
Step length (cm)	6.50	0.988 (0.981, 0.992)	0.71	1.97	<0.01
Stride length (cm)	12.88	0.962 (0.926, 0.980)	2.51	6.96	<0.01
Step width (cm)	3.54	0.987 (0.975, 0.994)	0.40	1.12	<0.01
Gait speed (km/h)	0.25	0.965 (0.932, 0.982)	0.05	0.13	<0.01

SD, standard deviation; ICC, intraclass correlation coefficient; 95% CI, 95% confidence intervals; SEM, standard error of measurement; MDC, the minimal detectable change at a 95% confidence level.

### The level of agreement

**FIGURE 2:**
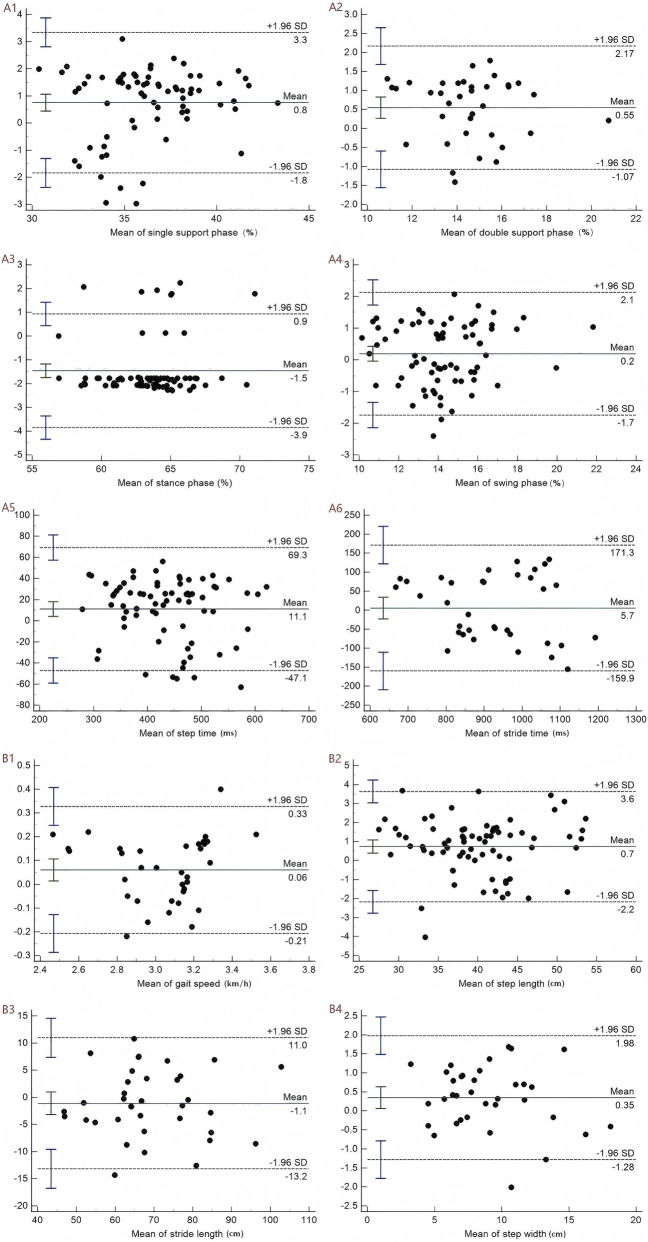
*Bland–Altman plots of gait indicators for inter-rater*. A represents the temporal parameters: A1 denotes the single-support phase, A2 the double-support phase, A3 the stance phase, A4 the swing phase, A5 the step time, and A6 the stride time. B represents the spatial parameters: B1 denotes gait speed, B2 step length, B3 stride length, and B4 step width.

**FIGURE 3:**
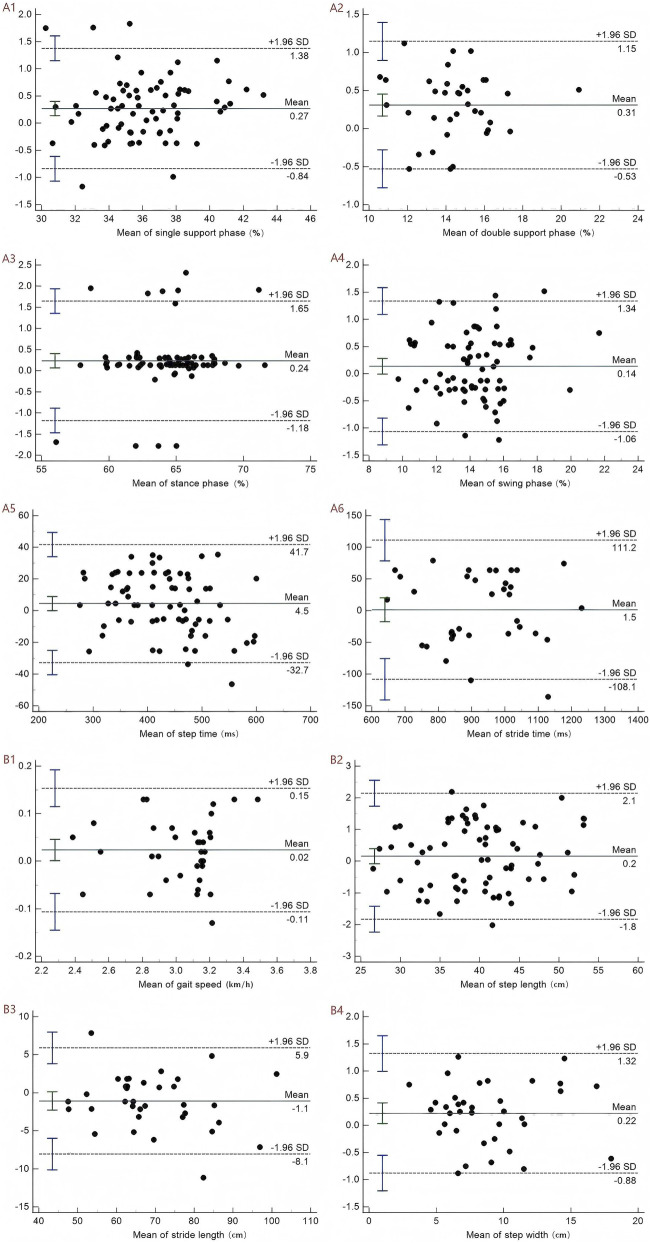
*Bland–Altman plots of gait indicators for test–retest*. A represents the temporal parameters: A1 denotes the single-support phase, A2 the double-support phase, A3 the stance phase, A4 the swing phase, A5 the step time, and A6 the stride time. B represents the spatial parameters: B1 denotes gait speed, B2 step length, B3 stride length, and B4 step width.

Figures 2, 3 showed Bland-Altman graphs illustrating the mean differences and 95% LOA for inter-rater and test–retest assessments of gait parameters. The mean differences ranged from −1.5 to 11.1 for inter-rater comparisons and from −1.5 to 4.5 for test–retest comparisons. The results indicated no substantial systematic bias was observed, and the agreement between inter-rater and test-retest was excellent across gait parameters.

### Muscle tone and stiffness of achilles tendon

Table 4 presented between-side differences in AT properties. The results indicated muscle tone and stiffness were significantly higher in the RSL compared with the RML (*P* < 0.05), as shown in Table 4.

**TABLE 4:** Difference between muscle properties between both sides (n = 36).

Variable	RSL	RML	MD	T Value	P Value	Cohen’s d value
Muscle tone (Hz)	26.19 ± 2.08	24.65 ± 1.93	1.53	2.921	0.006	0.487
Stiffness (N/m)	708.03 ± 54.16	641.50 ± 52.46	66.53	4.726	<0.001	0.788

RSL: relatively severe leg, RML: relatively moderate leg; MD: mean difference.

### The relationship between the asymmetry indexes of the achilles tendon properties and gait parameters, VAS score, and K/L grade

The results indicated that the Asy_Stiffness_ (AT) demonstrated a positive correlation with stance phase symmetry index (*ρ* = 0.514, P = 0.001), stride time (*ρ* = 0.381, *P* = 0.022), VAS score (*ρ* = 0.373, P = 0.025), and K/L grade (*ρ* = 0.542 *P* = 0.001), and a negative correlation with gait speed (*ρ* = −0.374, *P* = 0.025). No significant associations were observed between Asy_Stiffness_ (AT) and other gait parameters (*P* > 0.05). The Asy_Tone_ (AT) was not significantly correlated with gait parameters, VAS score, or K/L grade (*P* > 0.05), as shown in Table 5.

**TABLE 5:** Associations between Achilles tendon properties asymmetry indexes and gait parameters, VAS score, and K/L grade.

Variable	Asy_Tone_ (AT)		Asy_Stiffness_ (AT)	
	Spearman’s ρ	P -value	Spearman’s ρ	P -value
SI (stance phase) (%)	−0.258	0.129	0.514	0.001*
SI (single support phase) (%)	−0.141	0.411	−0.074	0.669
SI (swing phase) (%)	−0.025	0.883	−0.082	0.635
SI (step time) (ms)	0.025	0.886	0.082	0.633
SI (step length) (cm)	−0.076	0.658	−0.056	0.744
Double support phase (%)	0.103	0.550	0.035	0.839
Stride time (ms)	0.006	0.971	0.381	0.022*
Stride length (cm)	0.066	0.704	−0.022	0.900
Step width (cm)	0.242	0.155	−0.232	0.174
Gait speed (km/h)	0.290	0.087	−0.374	0.025*
VAS score	0.130	0.449	0.373	0.025*
K/L grade	0.152	0.376	0.542	0.001*

Asy_Tone_ (AT): asymmetry index of Achilles tendon muscle tone; Asy_Stiffness_ (AT): asymmetry index of Achilles tendon stiffness; SI, symmetry index; VAS, visual analogue scale; K/L, Kellgren/Lawrence; *p < 0.05.

### Independent factors associated with AT stiffness asymmetry index

Multivariate linear regression analysis was conducted to identify independent determinants of Asy_Stiffness_ (AT). Achilles tendon stiffness asymmetry index was specified as the dependent variable, with VAS score, K/L classification, SI (stance phase), stride time, and gait speed entered as independent variables. Dummy variable coding was performed for K/L classification, and K/L grade 1 was set as the reference group. After adjusting for age, sex, body weight, and ankle flexion range, the results indicated that stance phase symmetry index (*β′* = 0.298, *P* = 0.043), K/L grade 2 (*β′* = 0.533, *P* = 0.017) and K/L grade 3 (*β′* = 0.778, *P* = 0.002) remained independently associated with Asy_Stiffness_ (AT). VAS score, stride time, gait speed, and other K/L grades were not significant predictors (*P* > 0.05). The VIF values for all variables are below 5, as shown in Table 6.

**TABLE 6:** Independent factors associated with AsyStiffness (AT).

Variable	B	SE	β′	T Value	P Value	VIF
VAS score	0.778	0.576	0.172	1.350	0.188	1.268
SI (stance phase) (%)	0.614	0.289	0.298	2.122	0.043*	1.547
Stride time (ms)	0.009	0.006	0.177	1.452	0.157	1.167
Gait speed (km/h)	−5.331	3.769	−0.193	−1.415	0.168	1.459
K-L grade 2	7.461	2.927	0.533	2.549	0.017*	3.412
K-L grade 3	10.341	3.070	0.778	3.369	0.002*	4.171
K-L grade 4	9.089	5.355	0.226	1.697	0.101	1.388

AsyStiffness (AT): asymmetry index of Achilles tendon stiffness; B: unstandardized coefficient; SE: standard error; β': standardized coefficient; VIF: variance inflation factor; SI, symmetry index; VAS, visual analogue scale; K/L, Kellgren/Lawrence; *p < 0.05.

## Discussion

### Reliability of DIERS pedogait system

To our knowledge, this study is the first to confirm the reliability of DIERS pedogait system for assessing abnormal gait in KOA patients. The results demonstrated that DIERS pedogait system exhibits exceptionally high inter-rater and test-retest reliability in measuring the temporal and spatial parameters of gait in KOA patients.

We employed intraclass correlation coefficients (ICC) values to assess relative reliability, and results demonstrated that the inter-rater and test-retest reliability of each gait parameter fell within the excellent range, with ICC values ranging from 0.900 to 0.987 and 0.927 to 0.988, respectively. Notably, a study utilizing 3D gait analysis to assess patients with hip osteoarthritis found that spatial and temporal variables, along with most movement chains and trunk angles, exhibited good to excellent test-retest reliability (ICC values ranging from 0.77 to 0.97) (Laroche et al., 2011). Our findings are in alignment with those of the 3D gait analysis study, both demonstrating excellent reliability for gait assessment. DIERS pedogait system can therefore be considered a reliable tool for gait assessment. Additionally, a study using DIERS Formetric 4D to evaluate 40 spinal shape parameters at different time points in healthy adults reported that the majority of spinal shape parameters had excellent ICC values (greater than 0.75), with a few showing good to fair reliability (ICC between 0.40 and 0.75) (Degenhardt et al., 2020). These findings are consistent with our results. Variations observed in their study may be attributed to differences in participant characteristics such as age and BMI, as well as inconsistent measurement intervals.

The standard error of measurement (SEM) and the minimal detectable change (MDC) were employed to assess the absolute reliability of this study. SEM was used to quantify the consistency or degree of variation among sample means, while the MDC represented the smallest meaningful change that can be reliably interpreted (Furlan and Sterr, 2018). A higher ICC value corresponds to smaller SEM and MDC values, indicating greater absolute reliability (Chen et al., 2019). In both the inter-rater and retest analyses, the SEM values for all 10 gait parameters assessed by DIERS pedogait system ranged from 0.07 to 44.26 and 0.05 to 39.59, respectively, while the MDC values ranged from 0.20 to 122.68 and 0.13 to 109.74. Notably, the SEM and MDC values for stride time and step time were significantly higher than those for other variables. This may be attributed to two main factors: first, both stride time and step time are measured in milliseconds, resulting in larger measured values and wider standard deviation ranges compared to spatial parameters such as stride length and step width (measured in centimeters); second, temporal parameters, such as stride time and step time, are influenced by the stability of force exertion in the limbs of KOA patients and are closely linked to the force imbalance caused by joint pain (Mills et al., 2013). The variability of these temporal parameters may surpass that of spatial ones, such as step width. Additionally, we employed the Bland-Altman plot to calculate the differences between the two measurements and identify potential systematic biases in the dataset (Zidan et al., 2015). The results indicated that the distribution of mean differences between measurements did not exhibit significant systematic bias, with most values falling within the 95% confidence interval. The Bland-Altman plot’s distribution characteristics further validated the reliability of the measurement results in our study.

The Timed Up and Go test and the Stair Climb Test are widely used to assess gait impairment in KOA patients (Iijima et al., 2018; Holm et al., 2022), but they cannot directly quantify gait asymmetry or detailed spatiotemporal gait parameters. A three-dimensional motion analysis system might be the most appropriate approach, which can not only quickly obtain clinical information but also capture relevant temporal and spatial parameters (Broström et al., 2012). However, its high cost and operational complexity hinder its widespread use in clinical practice. DIERS pedogait system, with its excellent inter-rater and test-retest reliability, emerges as a promising alternative for assessing abnormal gait in KOA patients. It offers simplicity and non-invasiveness, delivering reliable results. This system delivers reliable gait parameters, including variations in stance phase, swing phase, step length and gait speed, and is suited to the diagnosis and management of foot and ankle disorders such as pes planus and pes cavus. Moreover, it can generate the trajectory of the plantar center of pressure, furnishing a scientific basis for customizing orthotic insoles and developing tailored rehabilitation plans for patients.

Reliable measurement of spatiotemporal gait parameters is critical for the effectiveness of asymmetry studies in KOA patients. Significant data variability can obscure true limb differences, leading to unreliable results (Laroche et al., 2011). Notably, DIERS pedogait system demonstrates excellent reliability in terms of inter-rater consistency and test-retest reliability, ensuring the comparability of asymmetry indices within this study cohort. Consistent with previous studies, we combined data from both the left and right lower limbs to enhance the statistical power of asymmetry analysis (Evans et al., 2003).

### Disparities in achilles tendon properties and their correlation with abnormal gait

Gait abnormalities and alterations in Achilles tendon properties in KOA patients are well established to be linked to biomechanical perturbations of the knee joint (Chen et al., 2021; Minea et al., 2025). Understanding the potential underlying relationship between gait abnormalities and Achilles tendon changes in KOA patients is crucial for both prevention and treatment strategies. Our study revealed that Achilles tendon muscle tone and stiffness in the RSL were significantly higher than those in the RML of KOA patients. Furthermore, correlation analysis demonstrated that the Asy_Stiffness_ (AT) was positively correlated with SI(stance phase), stride time, VAS scores, and K/L grade, while negatively correlated with gait speed. Multiple linear regression analysis further indicated that the SI(stance phase) alongside K/L grade 2 and 3 were independently associated with Asy_Stiffness_ (AT).

As expected, when classifying the RML and RSL based on pain intensity, persistent pain appears to induce changes in the biomechanical properties of the muscles. The discomfort within the affected knee joint may serve as a protective mechanism, reducing excessive load on the joint (Shen et al., 2025). However, prolonged overloading eventually leads to stiffness and atrophy of the surrounding muscles (Kaufman et al., 2001). Nazir et al. (Nazir and Ansari, 2024) have demonstrated a significant positive correlation between Achilles tendon thickness and the severity of KOA. Additionally, a comparative study investigating the relationship between KOA and Achilles tendon thickness revealed that the tendons of KOA patients were significantly thicker than those of healthy controls (Elbaz et al., 2017). Muscle stiffness, a key property of muscle tissue, reflects its ability to resist contraction or deformation under mechanical stress (Melo et al., 2022). An increase in muscle stiffness results in diminished muscle performance during movement (Kocur et al., 2019). Previous studies examining the properties of the Achilles tendon in patients with unilateral and bilateral KOA found that, regardless of whether the condition was unilateral or bilateral, the affected side exhibited significantly higher levels of muscle tension, stiffness, and elasticity compared to the non-affected side (Chen et al., 2021). Our findings align with this study, further supporting the notion that the functional properties of the Achilles tendon are intricately linked to the onset and progression of KOA. To mitigate knee joint loading, KOA patients reduce lower limb weight-bearing and flexion-extension loads. This adaptive behavior induces disuse atrophy of the triceps surae, a reduction in Achilles tendon cross-sectional area, disorganized collagen fiber alignment, and enhanced fiber adhesion (Liang et al., 2024). These changes may underpin the observed alterations in the stiffness of the Achilles tendon-associated musculature.

We employed an asymmetry index to characterize the interlimb imbalance in Achilles tendon properties among KOA patients (Chen et al., 2021). Correlation analyses revealed that the Asy_Stiffness_ (AT) exhibited a positive correlation with the stance phase symmetry index and stride time, as well as an inverse correlation with gait speed. The stance phase symmetry index, which represents the balance of stance phase durations between the two lower limbs, is such that higher values are associated with decreased symmetry in interlimb load distribution and stance phase timing (Arippa et al., 2022). From a biomechanical standpoint, relative elevation of Achilles tendon stiffness in the RSL substantially impairs its elastic deformability (Sinacore et al., 2013), compromising effective buffering of ground reaction force impacts on the knee joint during the stance phase (Du et al., 2023). To mitigate the risk of joint injury on the affected side, the body shortens the stance phase duration of the RSL to reduce its weight-bearing load, while prolonging that of the RML, thereby completing overall load transfer by extending the weight-bearing time of the RML (Delgado Caceres et al., 2021; Jover-Jorge et al., 2024). This differential adjustment in stance phase durations between the two sides ultimately manifests as a positive correlation between the Asy_Stiffness_ (AT) and the symmetry index of stance. Additionally, stride time correlates positively with Asy_Stiffness_ (AT), while gait speed correlates negatively with Asy_Stiffness_ (AT). Asymmetric Achilles tendon stiffness prolongs the coordinated force generation period of the bilateral ankle plantar flexor muscle tendon complexes (Machado et al., 2021), impairing the coordination of lower limb force production. The body maintains walking stability by extending the gait cycle and reducing gait speed (Smith et al., 2024), yet this compensatory adaptation further exacerbates the overall reduction in gait efficiency. Furthermore, we found that the Asy_Stiffness_ (AT) was positively associated with VAS scores and K/L grade, consistent with prior investigations (Chen et al., 2021). This may reflect that the pain and functional impairment caused by the progression of KOA further strengthened the protective gait adaptation, indirectly exacerbating the asymmetry in Achilles tendon stiffness (Falisse et al., 2022).

The multivariate linear regression analysis demonstrated that the stance phase symmetry index is an independent determinant of Asy_Stiffness_ (AT). Accordingly, correcting stance phase imbalance may help restore Achilles tendon function and potentially delay the progression of KOA. In addition, K/L grade 1 was set as the reference group, We found that K/L grade 2 and 3 were demonstrated as an independent factors associated with Asy_Stiffness_ (AT). This indicates that asymmetry in Achilles tendon stiffness is common in the early-mid stage of KOA. Notably, this cross-sectional study only confirms correlations between AsyStiffness (AT), gait parameters, and KOA severity, without establishing causal relationships among these variables. An alternative interpretation is that KOA patients initiate protective unloading strategies to avoid pain in the affected limb (Yalfani and Ahmadi, 2023). These strategies are characterized by shortened stance phase on the affected side, reduced ankle joint range of motion (Jandacka et al., 2017), and altered neuromuscular control during gait, and they may indirectly Achilles tendon properties. Regardless of whether Achilles tendon stiffness asymmetry represents a potential influencing factor or an adaptive consequence of KOA, implementing gait training to improve stance phase symmetry and Achilles tendon stretching exercises to mitigate stiffness asymmetry in early-mid stage patients may offer a viable approach for KOA prevention and management.

### Limations

There are several limitations in this study. First, although our study standardized treadmill speed and provided participants with a 10 min adaptation period, treadmill data may not fully reflect the gait of KOA patients during normal walking. Future research will compare gait parameters of KOA patients on the treadmill and over ground to validate the findings. Second, the sample size was modest with 36 KOA patients (72 lower limbs) enrolled. Certain subgroups, notably those with K/L grade 4, were statistically underpowered (n = 1), which may compromise the generalizability of correlation and regression results. Future studies will expand the sample size and increase the number of patients with K/L grades 1 and 4 to enhance the reliability of the results. Third, the study lacked matched healthy control subjects (including both non-obese and obese subgroups). Consequently, we could not validate the reliability of DIERS pedogait system for gait assessment in these two subgroups of healthy individuals, nor could we delineate differences in Achilles tendon properties or gait patterns between KOA patients and their respective healthy counterparts (both non-obese and obese). Future studies will incorporate stratified healthy control groups (non-obese and obese) to validate the system’s reliability in healthy populations and comprehensively characterize differences in Achilles tendon biomechanical properties and gait patterns between KOA patients and healthy non-obese or obese individuals. Fourth, this study adopted a cross-sectional design, which precluded establishing causal links between Achilles tendon properties asymmetry, gait abnormalities, and KOA progression. Future work will adopt a longitudinal approach to elucidate the sequential contributions of Achilles tendon stiffness asymmetry and gait abnormalities to disease advancement. Fifth, our study focused solely on spatiotemporal gait parameters in KOA patients and did not incorporate kinematic (e.g., joint angles, segment movements) or dynamic (e.g., joint torques, ground reaction forces) data. Both types of data are essential for understanding the biomechanical mechanisms underlying the relationship between Achilles tendon stiffness asymmetry and the progression of KOA. Future studies will integrate joint kinematic tools, such as 3D motion capture, to provide a comprehensive assessment of gait biomechanics in KOA patients.

## Conclusion

DIERS pedogait system demonstrated excellent inter-rater and test-retest reliability in assessing spatiotemporal gait parameters in KOA patients, providing a robust and objective tool for evaluating gait abnormalities. Additionally, muscle tone and stiffness of the Achilles tendon in the relatively severe leg were significantly higher than those in the relatively moderate leg in KOA patients. Notably, the Achilles tendon stiffness asymmetry index was significantly associated with abnormal gait parameters, VAS scores, and K/L grade. These findings suggest that early-mid interventions targeting gait abnormalities and reducing Achilles tendon stiffness asymmetry may provide novel strategies for the prevention and management of KOA.

## Data Availability

The original contributions presented in the study are included in the article/Supplementary Material, further inquiries can be directed to the corresponding authors.
