# Supplementary material for: Reliability of DIERS pedogait system for evaluating spatiotemporal gait parameters in knee osteoarthritis and its association with Achilles tendon stiffness asymmetry
Source: Front Physiol. 2026 Feb 11;17:1780014. doi: 10.3389/fphys.2026.1780014 (PMC12932186; doi:10.3389/fphys.2026.1780014)
Supplement: Supplementary file 1 [file DataSheet1.docx]

**Supplemental Appendix S1**

**Supplementary Figure 1**


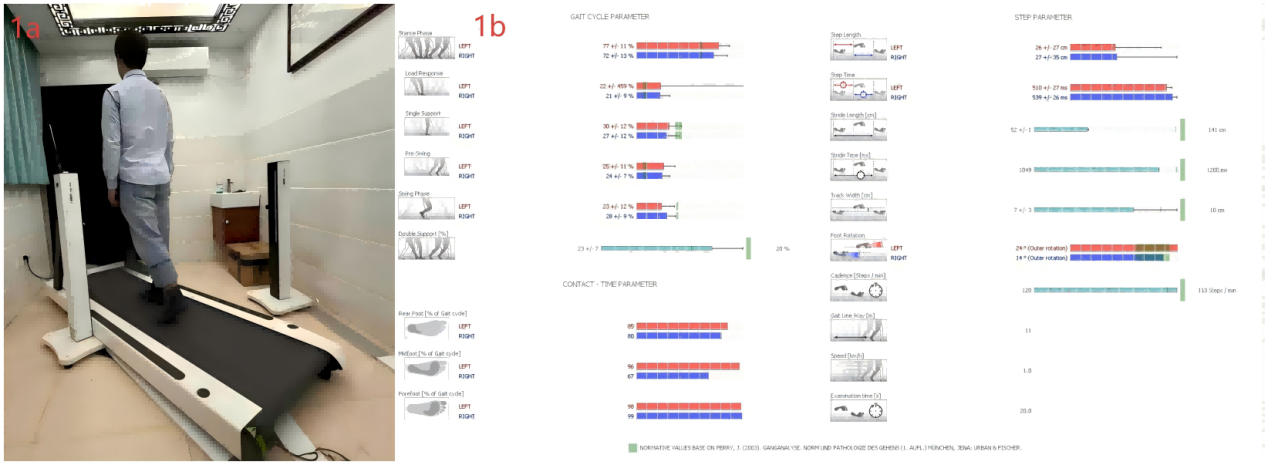


1a: The researcher employed DIERS pedogait to measure gait.

1b: Diagram of temporal parameters: single support phase (%), double support phase (%), stance phase (%), swing phase (%), stride time (ms), and step time (ms); (2) Spatial parameters: gait speed (km/h), step length (cm), stride length (cm), and step width (cm).

**Supplementary Figure 2**


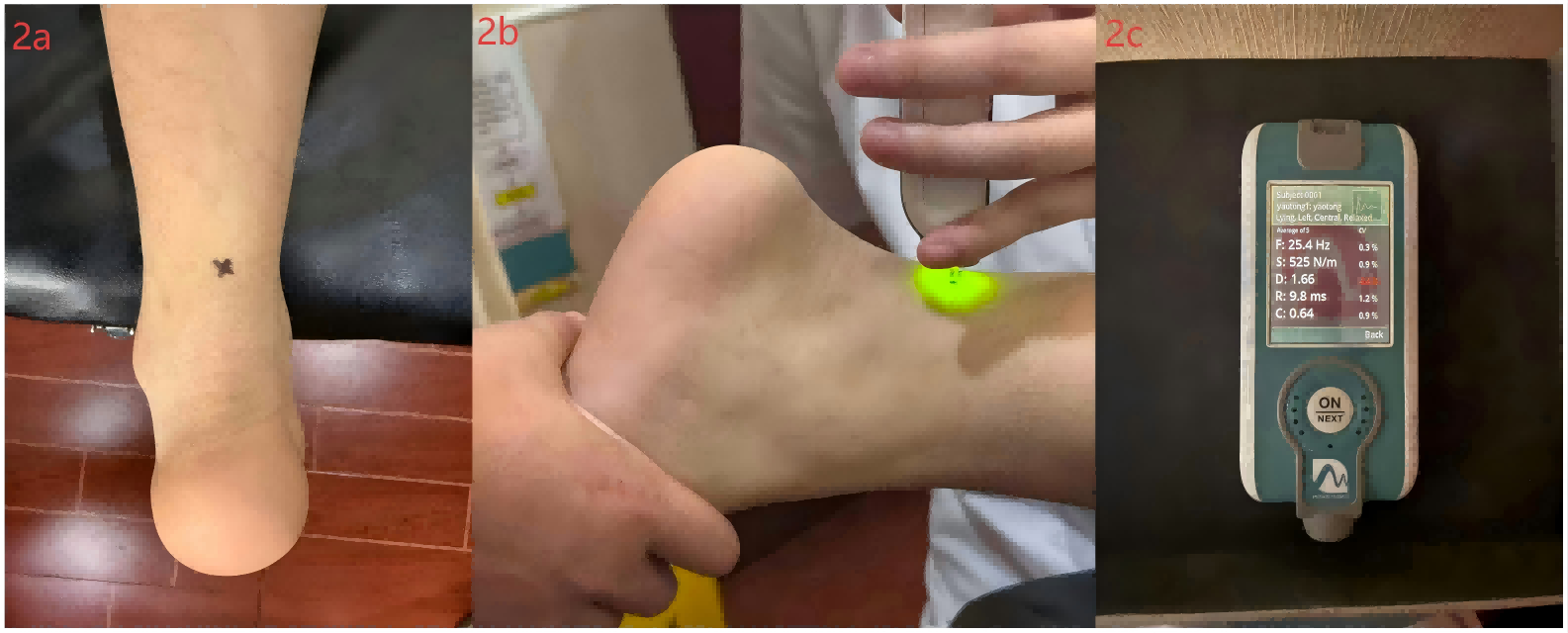


2a: The measurement position of achilles tendon with MyotonPRO

2b: The researcher employed MyotonPRO to conduct perform measurements.

2c: The data of MyotonPRO after measurement.

**Supplementary Method 1**

$Asy(\%)=\left( \frac{V_{larger}-V_{lower}}{V_{larger}} \right)\times100(\%)$

The muscle tone and stiffness asymmetry index of achilles tendon were calculated using this equation.

*Asy:* asymmetery index; *V_larger_*: larger value; *V_lower_*: lower value.

**Supplementary Table 1**

**The gait parameters collected by raters**

| **Variable** | **Rater 1** | **Rater 2** | **Rater 1*** |
| --- | --- | --- | --- |
| **Temporal parameters** |  |  |  |
| single support phase (%) | 35.94 ± 2.72 | 36.69 ± 2.91 | 36.21 ± 2.78 |
| double support phase (%) | 14.27 ± 2.05 | 14.82 ± 1.97 | 14.58 ± 2.06 |
| stance phase (%) | 64.25 ± 2.82 | 62.79 ± 2.91 | 64.48 ± 2.93 |
| swing phase (%) | 14.18 ± 2.15 | 14.37 ± 2.22 | 14.31 ± 2.21 |
| stride time (ms) | 921.90 ± 147.30 | 927.61 ± 132.22 | 923.44 ± 145.77 |
| step time (ms) | 430.58 ± 82.98 | 441.70 ± 79.16 | 435.08 ± 77.70 |
| **Spatial parameters** |  |  |  |
| step length (cm) | 39.70 ± 6.45 | 40.43 ± 6.46 | 39.86 ± 6.56 |
| stride length (cm) | 69.50 ± 13.25 | 68.41 ± 13.22 | 68.43 ± 12.49 |
| step width (cm) | 8.72 ± 3.53 | 9.08 ± 3.40 | 8.95 ± 3.54 |
| gait speed (km/h) | 3.14 (2.88 , 3.16) | 3.08 ± 2.53 | 3.13 (2.90 , 3.17) |

Rater 1*:Rater 1 conducted again 7 days later.
